# The Curcumin Analog CH-5 Exerts Anticancer Effects in Human Osteosarcoma Cells via Modulation of Transcription Factors p53/Sp1

**DOI:** 10.3390/ijms19071909

**Published:** 2018-06-29

**Authors:** Felipe Teixeira Lima, Viviane Seba, Gabriel Silva, Guilherme Silva Torrezan, Carlos Roberto Polaquini, Vitor Caressato Pinhanelli, Seung J. Baek, Ana Lúcia Fachin, Luis Octavio Regasini, Mozart Marins

**Affiliations:** 1 Biotechnology Unit, University of Ribeirão Preto, Av. Costábile Romano, 2201, Ribeirão Preto, SP CEP 14096-900, Brazil; lima.ft@hotmail.com (F.T.L.); vivianeseba@gmail.com (V.S.); biel-189@hotmail.com (G.S.); vitor.caressato@hotmail.com (V.C.P.); afachin@unaerp.br (A.L.F.); 2 Laboratory of Green and Medicinal Chemistry, Department of Chemistry and Environmental Sciences, Institute of Biosciences, Humanities and Exact Sciences, São Paulo State University (UNESP), São José do Rio Preto, SP CEP 15054-000, Brazil; guilherme_panz@hotmail.com (G.S.T.); carlos_polaquini@hotmail.com (C.R.P.); 3 Laboratory of Signal Transduction, College of Veterinary Medicine and Research Institute for Veterinary Science, Seoul National University, Seoul 08826, Korea; baeksj@snu.ac.kr; 4 Medicine School, University of Ribeirão Preto, Av. Costábile Romano, 2201, Ribeirão Preto, SP CEP 14096-900, Brazil

**Keywords:** osteosarcoma, curcumin, migration, invasion, apoptosis

## Abstract

Curcumin is a potential anticancer drug with poor bioavailability, which limits its clinical use as a therapeutic agent. The aim of this study was a preliminary evaluation of the curcumin analogue CH-5 as a cytotoxic agent in human osteosarcoma cell lines U2OS, MG-63, and Saos-2. CH-5 inhibited cell viability at lower concentrations than curcumin, leading to the induction of apoptosis. The cellular levels of the transcription factors p53 and Sp1 affect the expression of cellular pathways that lead to apoptosis. CH-5 increased p53 protein levels in U2OS cells and reduced Sp1 levels, with a consequent effect on the expression of their target genes DNA methyltransferase 1 (*DNMT1)* and growth arrest and DNA damage-inducible 45 alpha gene (*Gadd45a)*. CH-5 repressed *DNMT1* and increased *Gadd45a* mRNA expression, which was dependent on p53, as this effect was only observed in the colorectal cancer cell line HCT116 with active p53, but not in the isogenic p53-deficient HCT116 cells. CH-5 also reduced the protein levels of DNMT1, which led to the upregulation of *Gadd45a*. These results suggest that CH-5 has potentially higher anticancer activity than curcumin, which is associated with the expression of apoptosis-associated genes regulated by the transcription factors Sp1 and p53. Future work on CH-5 will define the therapeutic potential of this compound in vivo.

## 1. Introduction

Osteosarcoma (OS) is a rare but highly malignant cancer of the bone that occurs in any part of the skeleton and mostly affects children, adolescents, and young adults [1]. The current treatment includes local control surgery associated with multidrug systemic chemotherapy. A common chemotherapy regimen includes the combination of cisplatin, doxorubicin, and methotrexate for adjuvant and neoadjuvant therapy of localized and metastatic OS [2]. Despite the efficacy of these drugs, the very high propensity for local invasion and distant metastasis negatively affects the survival rate, which for metastatic patients is roughly 20% to 30% [3]. In these patients, multidrug resistance (MDR) is a major mechanism of resistance to chemotherapy that becomes an obstacle to successful treatment [4]. Furthermore, there are considerable side effects associated with the drugs used in OS chemotherapy, which include cardiotoxicity, nephrotoxicity, neurotoxicity, and induction of secondary neoplasia. Thus, more selective and effective chemotherapeutics are needed for OS.

The dietary phytochemical curcumin [(1E,6E)-1,7-bis(4-hydroxy-3-methoxyphenyl)-1,6-heptadiene-3,5-dione] is a constituent of turmeric powder derived from the rhizomes of *Curcuma longa*. It is used as a medicine in the treatment of inflammation, skin lesions, coughs, rheumatism, hepatic dysfunction, biliary dysfunction, sinusitis, and in the treatment of bacterial and fungal infections [5]. The broad spectrum of biological activities also includes cytotoxicity against tumor cells and inhibition of tumor growth [6], inhibition of angiogenesis and inflammation [7], as well as induction of apoptosis and cell cycle arrest [8]. It modulates the expression of genes involved in invasion, metastasis, angiogenesis, and resistance to chemotherapy [7,9,10]. These biological effects are associated with free radical scavenging properties and various molecular mechanisms, such as modulation of the expression of the Sp1 and p53 transcription factors and their target genes [9]. Moreover, epigenetic effects have been associated with the modulation of genes encoding epigenetic enzymes such as HDAC [11], HAT [12], and DNMT1 [13] and, more recently, with the modulation of microRNAs [14]. Its potential as a therapeutic agent for cancer has already been investigated for colorectal cancer, head, neck, pancreas, and osteosarcoma [15,16,17,18]. Although curcumin is considered a promising therapeutic agent for the treatment of different types of cancers, it is not physiologically stable and is not absorbed readily after ingestion [19]. This has driven the search for structural analogues with greater potency and bioavailability [20,21]. We have previously investigated the cytotoxic properties of a synthetic curcumin analog, 4,4′-[(2-Oxo-1,3-cyclohexanediylidene)-di(E)methylylidene] dibenzonitrile (named CH-5), in the human cancer gastric cell line HGC-27 [22]. In this cell line, CH5 reduced cell viability by apoptosis induction and decreased cell migration and invasion. In this study, we investigated the anticancer effects and possible molecular mechanisms underlying the effects of CH5 in OS cell lines. The results show that CH-5 is more potent than curcumin, and its anticancer activity could be mediated by the stabilization and downregulation of the transcription factors p53 and SP1, respectively, resulting in the activation of apoptosis by modulation of the expression of their target genes *DNMT1* and *Gadd45a.*

## 2. Results

### 2.1. CH-5 Inhibits Cell Viability of Osteosarcoma Cell Lines

**Figure 1:**
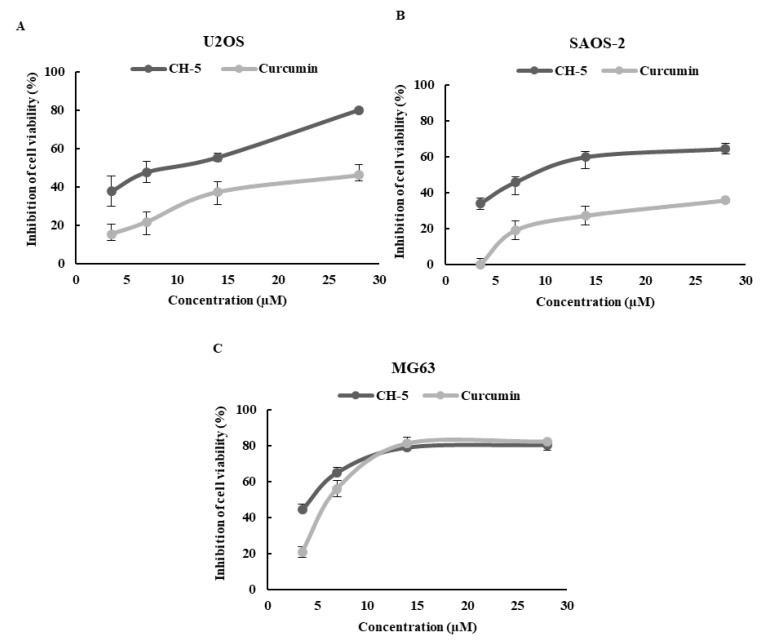
Inhibition of OS cell viability by CH-5 and curcumin was tested by the MTT assay. U2OS (**A**), Saos-2 (**B**), and MG-63 (**C**) cells were treated with various concentrations of curcumin and CH-5 for 24 h (control: cells treated with dimethyl sulfoxide). The results are expressed as mean ± standard deviation of three individual experiments having four technical replicates per experiment for each experimental condition.

We performed the [3-(4,5-dimethylthiazol-2-yl)-2,5-diphenyltetrazolium bromide] (MTT) reduction colorimetric assay to determine the effect of CH-5 and curcumin on cell viability of OS cell lines U2OS, Saos-2, and MG-63. CH-5 significantly reduced the viability of OS cells in a dose-dependent fashion in a 24 h treatment (Figure 1A–C). The IC50 values of curcumin at 24 h were found to be 27.7 + 3.2, 49.7 + 7.1, and 13.3 + 1.1 in U2OS, Saos-2, and MG-63 cells, respectively. The IC50 values of CH-5 in these cells were, respectively, 9.0 + 2.4, 11.7 + 2.4, and 4.4 + 0.7, indicating the higher potency of CH-5 over curcumin in the inhibition of cell viability (Table 1).

### 2.2. CH-5 Cytotoxicity Results in Apoptosis Induction

**Figure 2:**
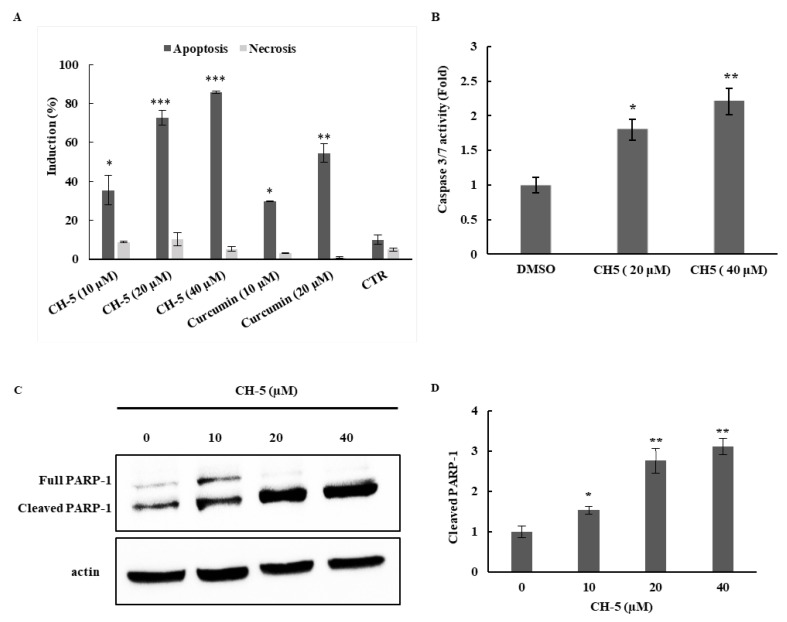
(**A**) Analysis of apoptosis and necrosis induction in U2OS cells treated with CH-5 and curcumin at the indicated concentrations for 24 h. The cells were double-stained with Annexin–FITC and PI to identify apoptotic and necrotic cells; (**B**) Apoptosis was further confirmed by measuring caspase 3/7 activity in U2OS cells treated with CH-5. After treatment with CH-5 at the indicated concentrations, the cells were collected and supplied with the substrate solution for caspase-3/7 activity determination, according to the manufacturer’s instructions, as described in materials and methods; (**C**) Western blot analysis of PARP-1 cleavage in U2OS cells treated with CH-5. U2OS were cultured and treated as described in 1A. Western blots of intact PARP-1 and cleaved PARP-1 in CH-5-treated and control (DMSO)-treated cells are shown; (**D**) The intensity of the PARP-1 fragment due to caspase activation was dose-dependent. The image intensities of the bands were normalized against the intensity of the β-actin band. The data are expressed as mean values ± SD (*n* = 3); * *p* < 0.05, ** *p* < 0.01 and *** *p* < 0.001 indicate a significant difference with respect to the control.

In order to determine whether the inhibition of CH-5 of the viability of OS cells was the result of apoptotic cell death, the externalization of phosphatidylserine (PS) during apoptosis was examined in U2OS cells by a fluorescence-conjugated annexin-V (FITC–AV)/propidium iodide (PI) double staining assay followed by flow cytometry. Annexin V is a 36 kDa calcium-binding protein with high affinity for PS, while the vital dye PI enters the dead and damaged cells that have compromised membranes but is excluded by the plasma membrane of viable cells. Therefore, viable cells are negative for both FITC–AV binding and PI, early apoptotic cells are positive for FITC–AV binding but negative for PI, late apoptotic cells are positive for both, whereas necrotic cells are negative for FITC–AV binding but positive for PI [23]. Approximately, 40% of U2OS cells were stained with FITC–AV after 24 h of exposure to 10 μM CH-5, about four times more than the control, treated with Dimethyl sulfoxide (DMSO), and two times more than cells treated with curcumin at the same concentration (Figure 2A). At a concentration of 20 μM, the number of apoptotic cells increased to almost 60% after curcumin exposure; however, once again, CH-5 was more potent, and more than 70% of cells were stained with FITC–AV at the same concentration. A further increase of CH-5 concentration to 40 μM elevated the number of apoptotic cells to over 80%, while the number of necrotic cells was low for all concentrations tested for both drugs. This dose-dependent fashion of apoptosis induction by CH-5 was also evident when assessing caspase 3/7 induction and cleavage of poly (ADP-ribose) polymerases-1 (PARP-1). Caspases are key effectors of apoptosis through the cleavage of various cellular substrates, such as pro-PARP-1 that is converted into the mature form PARP-1 [24]. As shown in Figure 2B, the treatment of U2OS cells with 20 and 40 μM CH-5 increased the levels of caspase 3/7 activity, as measured by a luminescence assay. Western blot analyses revealed a downregulation of the levels of pro-PARP-1 protein accompanied by an increase in the levels of PARP-1 protein, compared to the control (Figure 2C). Taken together, these results suggest that apoptosis induction by CH-5 is dependent on caspase activation in OS cells.

### 2.3. CH-5 Inhibits Cell Migration and Invasion

**Figure 3:**
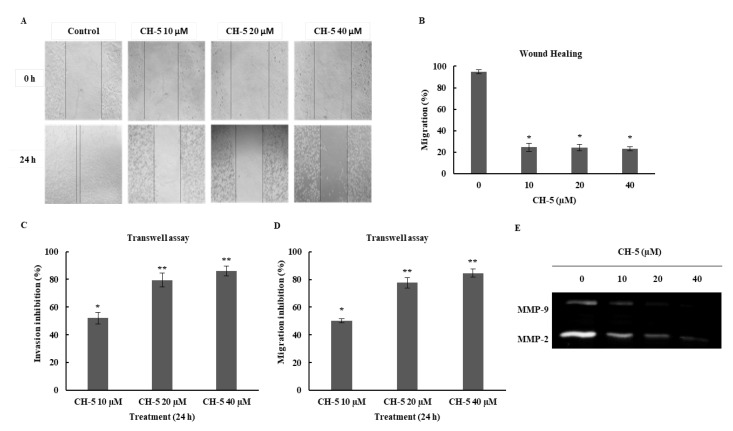
The effects of CH-5 on the migratory and invasive ability of U2OS cells. (**A**,**B**) U2OS migration in wound healing assays. A confluent monolayer was wounded with a sterile pipette tip, and the cells were allowed to migrate for 24 h in the presence of DMSO (control) or CH-5 at the indicated concentrations for 24 h. CH-5 reduced the migratory ability of U2OS cells compared to control cells (* *p* < 0.05); (**C**) Migration of U2OS cells in Transwell assays; (**D**) Invasion of U2OS cells in Matrigel, in Transwell assays. In both assays, the cells were seeded in the top chamber and treated with DMSO (control) or CH-5. After 24 h, the cells that had invaded through the membrane were stained, photographed, and quantified (bar graph). The data are expressed as means ± S.E.M. (*n*  =  3); * *p* < 0.05 and ** *p* < 0.01 vs. no treatment; (**E**) CH-5 suppresses the expression of matrix metalloproteinase MMP-2 and MMP-9 in U2OS cells. The cells were treated with CH-5 at the indicated concentrations for 24 h and then subjected to zymography to analyze the activity of MMP-2/-9.

A wound healing assay and a Transwell assay were conducted to investigate the motility of U2OS cells treated with CH-5 at 10, 20, and 40 μM. Compared with the control group, the wound healing assay showed that CH-5 significantly inhibited the migration of U2OS cells in a dose-dependent manner at 24 h (Figure 3A,B). The Transwell assay with or without Matrigel further demonstrated that, after 24 h of treatment with the same CH-5 concentrations, the migration activity and the invasive potential of U2OS was significantly reduced (*p* < 0.001 vs. no treatment) in a dose-dependent manner (Figure 3C,D). Furthermore, we examined by gelatin zymography analysis whether the inhibition of migration and invasion were accompanied by a decrease in the activity of metalloproteinases 2 and 9. These are two main metalloproteinases (MMPs) involved in the process of tumor cell invasion and metastasis. There was a reduction in the activity of both MMPs in CH-5-treated U2OS cells compared to control cells, in a dose-response manner (Figure 3E). These results clearly suggest that CH-5 possesses an anti-migratory and anti-invasive effect in U2OS cells.

### 2.4. CH-5 Increases p53 and Reduces Sp1 Protein Levels in U2OS Cells

**Figure 4:**
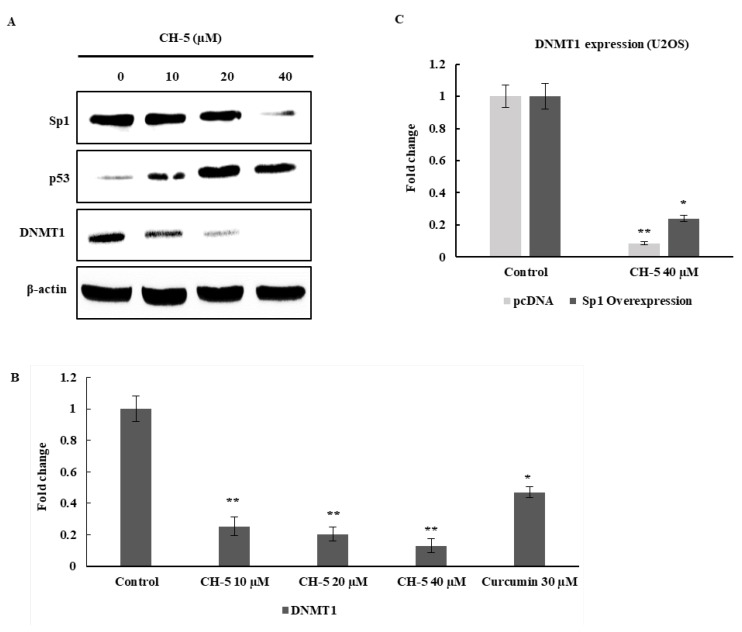
(**A**) CH-5 affects the expression of Sp1, p53, and DNMT1 proteins in U2OS cells. The cells were grown in a 60 mm dish and then were incubated with CH-5 at the indicated concentrations for 24 h. A 30 µg aliquot of total proteins was examined by western blotting, as described in Materials and Methods; (**B**) Effect of CH-5 and curcumin on the expression of DNMT1 mRNA, assessed by RT-PCR. U2OS cells were treated with DMSO, CH-5 10, 20, and 40 µM, or curcumin 30 µM for 24 h. The transcript levels were normalized using RPL30 as a reference gene; * *p* < 0.05 and ** *p* < 0.01; (**C**) Effects of CH-5 treatment and Sp1 overexpression on the mRNA levels of DNMT1. When U2OS cells were simultaneously exposed to CH-5 (40 µM) and transfected with an Sp1-expressing vector, there was a weaker downregulation of DNMT1, compared to control cells transfected with a control empty vector. The data are indicated as fold change in relative expression compared with RPL30 as a reference gene and on the basis of the comparative ΔΔ*C*_t_ method, and expressed as the mean ± standard deviation; * *p* < 0.05 and ** *p* < 0.01.

The transcription factors p53 and Sp1 regulate various cell functions, including the promotion of apoptosis, suppression of cell growth, migration, and invasion [25,26,27]. To further investigate the underlying molecular mechanisms of CH-5-mediated anticancer activities, the expression level of p53 and Sp1 proteins was examined in U2OS cells treated with CH-5, using Western blotting analysis. Sp1 was downregulated, and p53 was upregulated following CH-5 treatment, in a dose-dependent manner (Figure 4A).

### 2.5. The Effect of CH-5 on the p53/Sp1 Axis Affects the Expression of DNA Methyltransferase (DNMT1) Gene

DNMT1 is the primary enzyme responsible for the maintenance of DNA methylation in genomic DNA, and the disruption of its function causes chromosome instability and dysregulation of transcription and ultimately leads to p53-dependent apoptotic cell death [28]. The levels of p53 and Sp1 proteins determine the promoter activity of *DNMT1* [29] and of other target genes [27]. Using quantitative real-time PCR and western blot analysis, we found that CH-5 downregulated DNMT1 protein expression and mRNA in the U2OS cells in a dose-dependent manner (Figure 4A,B). Sp1 is a transcription activator for the *DNMT1* gene [30], and overexpression of Sp1 in U2OS cells reduces the suppressive effect of CH-5 on the expression of *DNMT1* (Figure 4C).

### 2.6. CH-5 Modulation of Gene Expression is p53-Dependent

**Figure 5:**
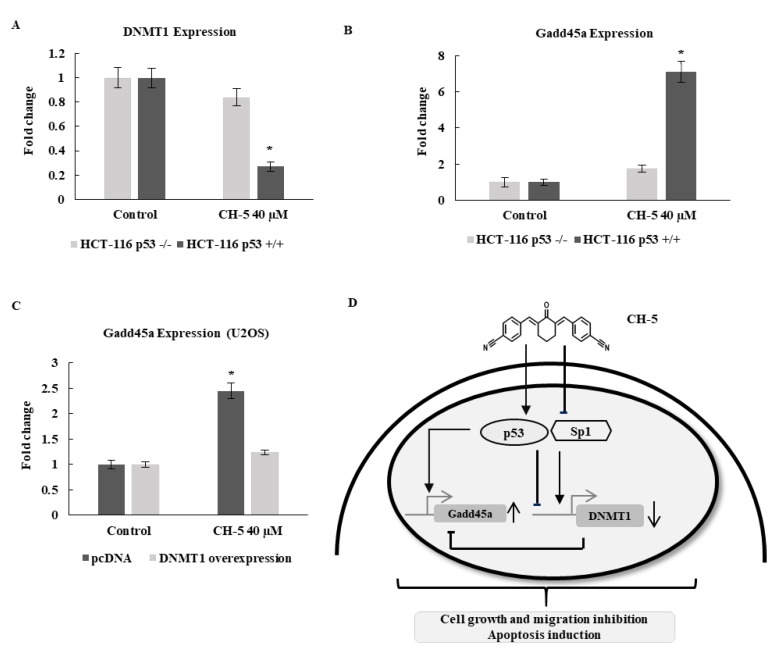
(**A**) Downregulation of DNMT1 (**A**,**B**) and upregulation of *Gadd45a* were observed in HCT116 wild-type (WT) cells treated with CH-5 (40 µM) for 24 h, but not in HCT116 p53^−/−^ cells; (**C**) *Gadd45a* mRNA expression is reduced when U2OS cells are simultaneously exposed to CH-5 (40 µM) and transfected with a DNMT1-expressing vector, compared to control cells transfected with an empty vector. The data are indicated as fold change in relative expression compared with RPL30 as a reference gene and on the basis of the comparative ΔΔ*C*_t_ method, and expressed as the mean ± standard deviation; * *p* < 0.05; (**D**) A proposed model for CH-5’s action in osteosarcoma cells. CH-5 up- and downregulated p53 and Sp1 protein levels, respectively, thus promoting *DNMT1* repression and then *Gadd45a* induction.

We used the isogenic human colon carcinoma cell lines HCT116 p53^+/+^ and HCT116 p53^−/−^ to investigate the effect of p53 on CH-5 inhibition of endogenous DNMT1 expression. Quantitative RT-PCR analysis of *DNMT1* mRNA levels in these cell lines revealed a 1.9-fold reduction in the level of *DNMT1* mRNA in the p53^+/+^ cells, while no significant reduction was observed in the p53^−/−^ cells (Figure 5A). The growth arrest and DNA damage-inducible 45 alpha gene (*Gadd45a*) is another p53 target and a stress-inducible gene implicated in DNA repair and apoptosis; more recently, it has also been implicated in inhibition of cell invasion and migration [31,32]. We also determined by qRT-PCR the mRNA expression level of *Gadd45a* in the HCT p53^+/+^ and p53^−/−^ cells treated with CH-5. As shown in Figure 5B, a 2.95-fold induction of *Gadd45a* mRNA expression was observed in HCT116 p53^+/+^ in response to CH-5 treatment, whereas in p53^−/−^ cell, there was only a 0.81-fold induction. Taken together, these results suggest that the inhibition of *DNMT1* and activation of *Gadd45a* by CH-5 is p53-dependent.

### 2.7. DNMT1 Overexpression Diminishes CH-5-Induced Gene Activation of Gadd45a

The *Gadd45a* gene is inactivated by promoter DNA methylation in various types of cancer and tumor cell lines. Treatment of U2OS cells with the DNMT1 inhibitor decitabine activates *Gadd45a* mRNA expression and leads to apoptosis [33]. In order to see if the induction of *Gadd45a* mRNA expression by CH-5 could be linked to DNMT1 inhibition, we overexpressed DNMT1 in U2OS cells and measured Gadd45a mRNA expression by quantitative PCR. The induction of *Gadd45a* mRNA expression by CH-5 was lower in U2OS overexpressing DNMT1 than in control cells transfected with the empty vector (Figure 5C). This result suggests that CH-5 induction of *Gadd45a* is mediated by DNMT1 inhibition and possibly by its promoter demethylation.

## 3. Discussion

Many in vitro and in vivo studies describe curcumin as a promising anticancer drug [15,16,17,18], but its instability and poor metabolic properties limit its clinical application [19]. Therefore, curcumin analogues are frequently investigated for improved chemical properties that still maintain its anticancer activity [21,34].

Several reports describe the antiproliferative and apoptosis-inducing activity of curcumin in different cancer cell lines [8,12,13,14,16,18]. Modified curcumin analogues with replacement of the α,β-unsaturated monoketone with the α,β-unsaturated diketone of curcumin have been reported to have better pharmacokinetic profiles and enhanced anticancer activity [35,36]. In the present study, we tested the cytotoxicity of the monoketone curcumin analogue CH-5 in human OS cell lines U2OS, MG-63, and Saos-2. The MTT assay results indicated powerful cytotoxic effects in these cell lines in a dose-dependent manner and with higher potency than curcumin. We observed by flow cytometry, ELISA, and western blot assays that CH-5 cytotoxic activity was due to the induction of caspase-dependent apoptosis, which was also higher than in curcumin-treated cells. It has been reported that the α,β-unsaturated ketone moiety of curcumin can react via Michael nucleophilic addition with the thiol group of intracellular glutathione (l-γ-glutamyl-l-cysteinyl-glycine, GSH) [37] and of Thioredoxin NADPH Reductase (TrxR) that play a key role in maintaining cellular redox balance [38]. A reduction in cellular GSH levels and the inhibition of TrxR activity affect protection against oxidative stress, which can lead to apoptosis [39,40]. We suggest that the α,β-unsaturated ketone moiety of CH-5 could also contribute to apoptosis induction by a similar mechanism.

We have also found that CH-5 inhibited OS cell migration and invasion, as confirmed by scratch wound healing and Transwell migration assays. Migration and invasion are important events in cancer progression, especially in metastasis [41], which is an important aspect of OS malignancy and accounts for high mortality [42]. Matrix metalloproteinases 2 and 9 (MMP-2 and MMP-9) are members of a family of zinc-dependent endopeptidases and contribute to the ability of tumor cells to degrade extracellular matrix components during tumor cell growth and metastasis [43,44]. In this study, we observed a concentration-dependent decrease in secreted MMP-2 and MMP-9 protein levels in U2OS cells treated with CH-5. This result indicates that the anti-migration and anti-invasion effect of CH-5 is conferred, at least in part, by inhibition of MMP secretion.

Apart from the effects resulting from the direct binding to effector target molecules, the anticancer effects of curcumin and its analogues can also involve transcriptional regulatory pathways responsible for the regulation of genes involved in tumorigenesis, angiogenesis, metastasis, invasion, proliferation, and apoptosis [9,10]. Various reports demonstrated the involvement of the transcription factors p53 and Sp1 in the effect of curcumin over these pathways in various cancer cells [45,46]. The treatment of U2OS cells with CH-5 caused the upregulation of p53 protein and the downregulation of Sp1 protein. This effect was reflected also in the expression of these transcription factors’ target genes *Gadd45a* and *DNMT1*. *DNMT1* gene expression is repressed by a p53/Sp1 complex bound to the promoter of the gene, but a low level of Sp1 enhances the repressive activity of p53, whereas high levels upregulate *DNMT1* gene expression [29]. In accordance with these data, treatment of U2OS cells led to an inhibition of *DNMT1* expression, dependent on p53, as demonstrated with the p53-null HCT cells. Moreover, the repression of *DNMT1* by CH-5 could be reduced by overexpression of Sp1. *Gadd45a* gene is a DNA damage-inducible gene that participates in p53-dependent apoptosis [47] and is upregulated by activated p53 [48]. The expression of *Gadd45a* could be induced in p53 wild-type HCT116 cells treated with CH-5, but not in p53-null HCT116 cells. A methylation-mediated repression of *Gadd45a* expression has been described in U2OS cells and could be inhibited by treatment with DNMT1 inhibitors [33]. Interestingly, the overexpression of DNMT1 abrogated induction of Gadd45a mRNA expression by CH-5 in U2OS cells. Taken together, these results indicate that CH-5 can indirectly affect the expression of genes by the modulation of transcription factors and possibly by epigenetic mechanisms.

## 4. Materials and Methods

### 4.1. Synthesis of the Curcumin Analogue CH-5

**Figure 6:**
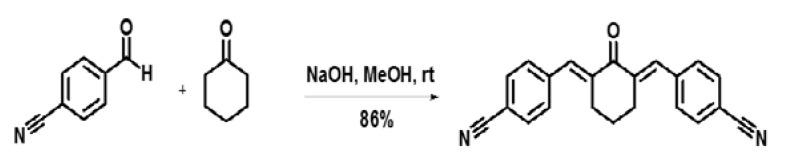
Synthesis of CH-5.

The synthesis of CH-5 was achieved by aldol condensation, using the general procedure reported by Hassan and co-authors, with minor modifications [49]. Briefly, 5 mL cyclohexanone (Sigma-Aldrich^®^, St. Louis, MO, USA) was added to a solution of 10 mL 4-formylbenzonitrile (Sigma-Aldrich^®^) in methanol (10 mL). The solution was stirred at room temperature for 10 min, followed by dropwise addition of a methanolic solution of NaOH (0.5 mL, 1.0 M). The reaction medium was stirred at room temperature and monitored by successive TLC analyses. When the reaction was finished, the residue was poured into crushed ice, resulting in precipitate, which was removed by filtration, washed with cold water, and recrystallized in ethanol. After these procedures, CH-5 was obtained in 86% yield (Figure 6). The structure of the compound was identified by 1H NMR and 13C NMR spectra analyses (Supplementary Materials).

### 4.2. Cell Culture

Human OS cell lines U2OS (p53 wt), Saos-2 (p53 null), and MG-63 (p53 mut) were purchased from the Rio de Janeiro Cell Bank (BCRJ, Federal University of Rio de Janeiro, Rio de Janeiro, Brazil). HCT116 p53^+/+^ and HCT116 p53^−/−^ cells were gently provided by Dr. Bert Vogelstein (John Hopkins University Howard Hughes Medical Institute and Kimmel Cancer Center, Baltimore, MD, USA). U2OS and MG-63 cells were grown in McCoy’s 5A Medium (Sigma-Aldrich^®^), while Saos-2 and HCT166 cells were grown in Dulbecco’s Modified Eagle Medium (Sigma-Aldrich^®^), supplemented with 10% fetal bovine serum (FBS), 100 U/mL penicillin, and 100 µg/mL streptomycin (Sigma-Aldrich^®^). The cells were cultured at 37 °C in a humidified atmosphere of 5% CO_2_, for all experiments.

### 4.3. Cell Viability Assay

Cell viability was measured by the MTT assay. In brief, the cells were seeded in 96-well plates at a density of 5 × 10^3^ cells/well. After overnight culture, the cells were treated in quadruplicate with 0, 1.25, 2.5, 5.0, and 10 µg/ml of CH-5 and curcumin for 24 h. After treatment, the medium was replaced with fresh medium, 20 µL of an MTT [3-(4,5-dimethylthiazol-2-yl)-2,5-diphenyltetrazolium bromide] (Sigma-Aldrich^®^) solution (5 mg/mL) was added to each well, and the plates were incubated for an additional 3 h. The absorbance was measured spectrophotometrically at a wavelength of 550 nm by a microplate reader (MultiSkan FC, Thermo Scientific, Waltham, MA, USA). The results were plotted as percentage of inhibition of cell viability (ICV) calculated as follows: ICV (%) = [1 − (absorbance of treated group/absorbance of control group)] × 100. The data represent the average ± SD from three independent experiments combined.

### 4.4. Apoptosis Assay

The determination of apoptotic cells by flow cytometry was performed by double labeling (Annexin-V/propidium iodide), using an FITC–Annexin V Apoptosis Detection Kit I (BD Biosciences, San Jose, CA, USA). Briefly, U2OS cells were cultured in 6-well plates (7.5 × 10^5^ cells/well), followed by treatment with 0, 10, 20, and 40 µM CH-5 in a medium with 10% FBS for 24 h. The adherent and floating cells were collected together, and an apoptosis assay was conducted according to the manufacturer’s protocol. The cells were examined using BD FACSCalibur™ Flow Cytometer (BD Biosciences).

### 4.5. Caspase 3/7 Assay

The activity of caspase 3/7 was evaluated using the Apo-ONE Homogenous Caspase-Glo 3/7 Assay (Promega, Madison, WI, USA). U2OS cells grown in 60 mm plates were treated with 20 or 40 µM CH-5 in a medium with 10% FBS for 24 h. Subsequently, protein lysates were obtained in passive lysis buffer (Promega) containing protease and phosphatase inhibitors. Amounts of total proteins (30 µg) were distributed into black-walled 96-well plates and mixed with the same volume of caspase–Glo 3/7 reagent. After incubation at room temperature in the dark for 1 h, the luminescence was measured in an FL 800 microplate reader (Bio-Tek Instruments, Winooski, VT, USA).

### 4.6. Wound Healing Assay

A wound healing assay was performed to analyze the cells’ migration capacities. Briefly, U2OS cells were grown in 24-well plates until the cell monolayer reached nearly 100% confluency; then, a wound was created with a sterile 10 μL pipette tip. The floating cells were removed, and the attached cells were exposed to the indicated concentration of CH-5 for 0 and 24 h. At the end of the incubation, the scratched areas were photographed in an optical microscope. The wound areas were measured using ImageJ software (ImageJ2, National Institutes of Health, Bethesda, MD, USA). The migration rates of CH-5-treated and DMSO-treated cells were calculated as follows: migration rate (%) = [(wound area at 0 h − wound area at 24 h)/wound area at 0 h] × 100%.

### 4.7. Transwell Assay

A Transwell cell assay was used to analyze the effect of CH-5 on the migration and invasion capacity of U2OS cells. For the migration assay, the cells were serum-starved overnight; then, 2×10^5^ cells resuspended in 200 µL serum-free medium were seeded into the upper chamber of 24-well Transwell plates of 8 µm pore size (Corning^®^, Kennebunk, ME, USA). Into the lower chamber, 750 µL of complete medium (with 10% serum), containing different concentrations of CH-5 was added. After treatment for 24 h, the migrated cells attached on the bottom side of the Transwell membrane were fixed in 3.7% of paraformaldehyde for 2 min, permeabilized with methanol 100%, and stained with Wright’s Giemsa solution for 15 min at room temperature. For the invasion assay, the membranes of the Transwell were pre-coated with 0.75 mm of Matrigel (Corning^®^) according to the manufacturer’s instructions, and then the same procedure as for the migration assay was performed. After migration or invasion, the cells were destained with 100 µL of cells of 33% acetic acid. The destaining solution was collected, and the absorbance was measured at a wavelength of 490 nm by a microplate reader (MultiSkan FC, Thermo Scientific). The inhibition of migration or invasion was calculated as follows: Inhibition (%) = [1 − (absorbance of treated group/absorbance of control group)] × 100. The data represent the average ± SD from three independent experiments.

### 4.8. Reverse Transcription-Polymerase Chain Reaction (RT-PCR)

RT-PCR was performed to analyze gene expression according to previous protocols. In summary, U2OS cells were seeded in 6-well plates at a density of 5×10^5^ cells/well and cultured overnight. Next, the cells were treated with 0, 10, 20, and 40 µM of CH-5 in serum-free medium for 24 h. After treatment, total RNA was isolated using IllustraTM RNASpin Mini Kit (GE Healthcare, Little Chalfont, UK) and treated with DNase I Amplification Grade (Sigma-Aldrich^®^). Then, 1 µg of RNA was used for the synthesis of cDNA using the High-Capacity cDNA Reverse Transcription Kit (Applied Biosystems). The PCR was carried out using the TaqMan^®^ Gene Expression Assay (Applied Biosystems, Foster City, CA, EUA ) for the genes of interest (DNMT1-ID: Hs00154749_m1 and Gadd45a-ID: Hs00169255_m1), according to the manufacturer’s instructions. The amplification reactions were carried out in triplicate in an Mx3005P real-time thermocycler (Stratagene, La Jolla, CA, USA), with one hold at 95 °C for 10 min, and 40 cycles at 95 °C for 15 s and 60 °C for 1 min. The RPL30 constitutive gene (ID: Hs00265497_m1) was used as a reference gene for normalization of the results, and the fold-differences in gene expression were calculated relative to the DMSO-treated samples using the ΔΔ*C*_t_ method.

### 4.9. Gelatin Zymography

Gelatin zymography was performed to quantify matrix metalloproteinase-2 and 9 (MMP-2 and MMP-9) activity. Briefly, U2OS cells were cultured in 6-well plates (5 × 10^5^ cells/well) followed by treatment with 0, 10, 20, and 40 µM of CH-5 in serum-free medium for 24 h. After treatment, the supernatant (conditioned medium) from each sample was collected and then separated by 0.1% gelatin-7% SDS-PAGE electrophoresis. Next, the gels were washed in 2.5% Triton X-100 for three times (30 min each) at room temperature and incubated in the reaction buffer (10 mM CaCl2, 40 mM tris-HCl and 0.01% NaN3, pH 8.0) at 37 °C for 18 h. The gels were washed with distilled water and stained with Coomassie brilliant blue R-250. After destaining, the bands were digitally scanned, and the gelatinolytic activities were measured using ImageJ software.

### 4.10. Western Blot

U2OS cells were cultured in 60 mm dishes (70–80% confluence), followed by treatment with 0, 10, 20, and 40 µM of CH-5 in serum-free medium for 24 h. Next, protein lysates were obtained in RIPA buffer supplemented with proteinase inhibitors. Amounts of total proteins (30 µg) were separated on 12% SDS-PAGE gel, and transferred onto nitrocellulose membranes (Pall Corporation, Pensacola, FL, USA). The membranes were blocked in tris buffered saline with Tween 20 (TBST) (25 mM Tris, 3 mM KCI, 0.14 M NaCl, and 0.05% Tween 20), containing 5% nonfat milk, at room temperature for 1 h. Next, the membranes were incubated in TBST-5% nonfat milk, containing the primary antibodies (1:1000) overnight at 4 °C. After washing with TBST, the membranes were incubated with horseradish peroxidase-conjugated secondary antibodies diluted in TBST-5% nonfat milk (1:10,000) for 1 h and washed several times. The proteins were detected by chemiluminescence, using the ECL Western Blotting Detection Reagent (Amersham Biosciences, Piscataway, NJ, USA) in a luminescence analyzer LAS4000 (Fujifilm Medical Systems, Stamford, CT, USA).

### 4.11. Expression Vector and Transfection

The expression vector pcDNA3/Myc-DNMT1 was a gift from Arthur Riggs (Addgene plasmid # 36939) [50]. The full-length Sp1 expression vector [51] and pcDNA empty vector (Invitrogen, Grand Island, NY, USA) were previously described. The transient transfections were carried out using Lipofectamine 3000 Reagent (Thermo Scientific, Rockford, IL, USA), according to the manufacturer’s protocol. U2OS cells grown in 6-well plates were transfected with 3 µg of Sp1 and DNMT1 expression vector or 3 µg of pcDNA empty vector. After 48 h from the start of the transfection, the cells were treated with 40 µM CH-5 in serum-free media for 24 h. After treatment, the cells were collected, RNA was isolated, and qPCR was performed.

### 4.12. Statistical Analysis

The statistical analysis was carried out with one-way ANOVA test followed by a Tukey’s HSD test. The results were considered statistically significant if * *p* < 0.05, ** *p* < 0.01, and *** *p* < 0.001.

## 5. Conclusions

In summary, our study reported a curcumin analogue CH-5, which exhibited anticancer activity against OS cell lines. CH-5 was more potent than curcumin in the inhibition of proliferation and in the induction of apoptosis. It induced stabilization of p53 and repression of Sp1 proteins, with a consequent effect on the expression of their target genes. CH-5 repressed the expression of *DNMT*1 gene, which released the expression of the proapoptotic gene *Gadd45a* (Figure 5D). Therefore, CH-5 is a promising lead compound in the treatment of OS, but further studies are needed to clarify its exact mechanism, possibly involving research on the inhibition of DNA methylation, on the compound’s bioavailability, and on its antitumor effects in vivo.

## Figures and Tables

**Table 1:** IC50 (µM) values after treatment with curcumin and CH-5 for 24 h.

Cells	CH-5	Curcumin
U2OS	9.0 ± 2.4	27.7 ± 3.2
Saos-2	11.7 ± 2.4	49.7 ± 7.1
MG-63	4.4 ± 0.7	13.3 ± 1.1

## Supplementary Materials

The following are available online at http://www.mdpi.com/1422-0067/19/7/1909/s1.
