# Supplementary material for: The Curcumin Analog CH-5 Exerts Anticancer Effects in Human Osteosarcoma Cells via Modulation of Transcription Factors p53/Sp1
Source: Int J Mol Sci. 2018 Jun 29;19(7):1909. doi: 10.3390/ijms19071909 (PMC6073932; doi:10.3390/ijms19071909)

Figure S.1. Curcumin analogue CH-5

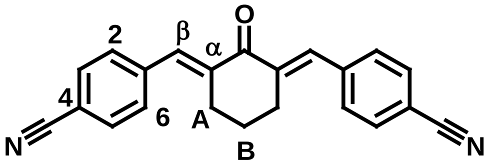

CH-5

^{1}H NMR (600 MHz), δ_H (ppm): 1.82−1.87 (m; H-B), 1.92−1.94 (m; H-A), 7.55 (d; 8.4 Hz; H-3 and H-5), 7.72 (d; 8.4 Hz; H-2 and H-6), 7.77 (s; H-β).

^{13}C NMR (150 MHz), δ_C (ppm): 22.6 (C-B), 28.3 (C-A), 112.0 (C-4), 118.6 (4-**CN**), 130.6 (C-2 and C-6), 132.2 (C-3 and C-5), 135.2 (C-β), 138.3 (C-α), 140.2 (C-1), 189.4 (C=O).

Figura S.2. ^{1}H NMR spectrum of compound **CH-5** ($CDCl_3$, 600 MHz)

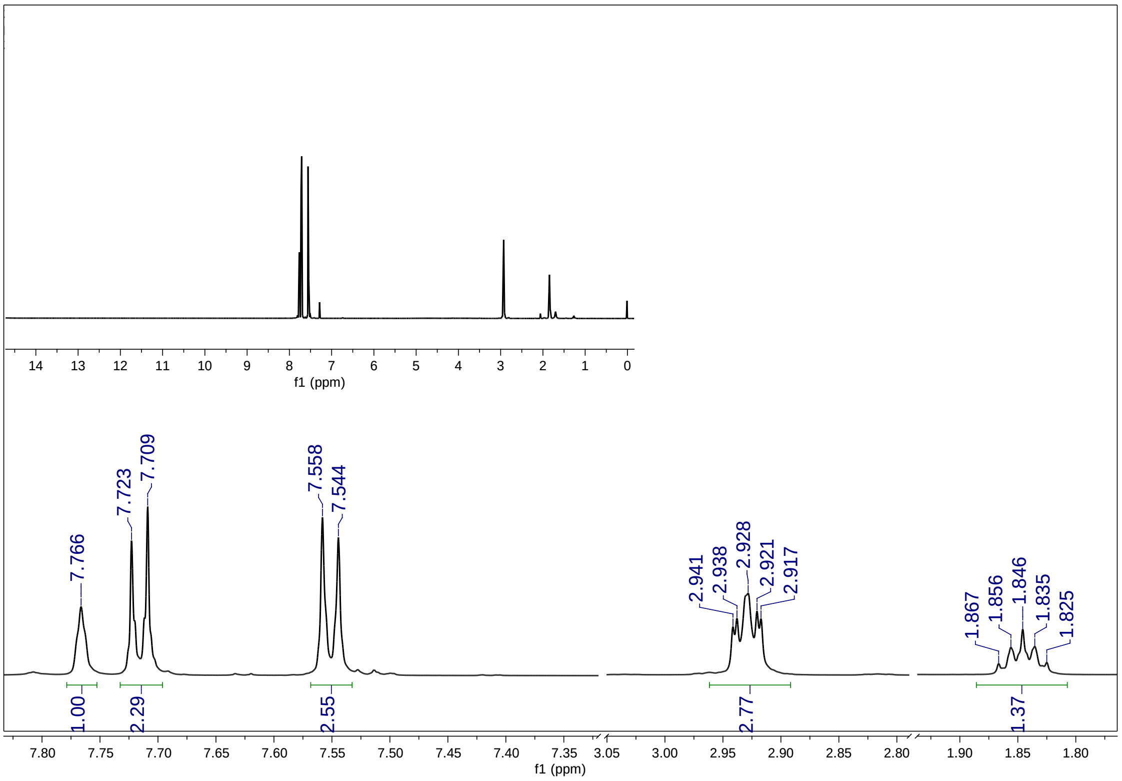

Figura S.3. ^{13}C NMR spectrum of compound **CH-5** ($CDCl_3$, 150 MHz)

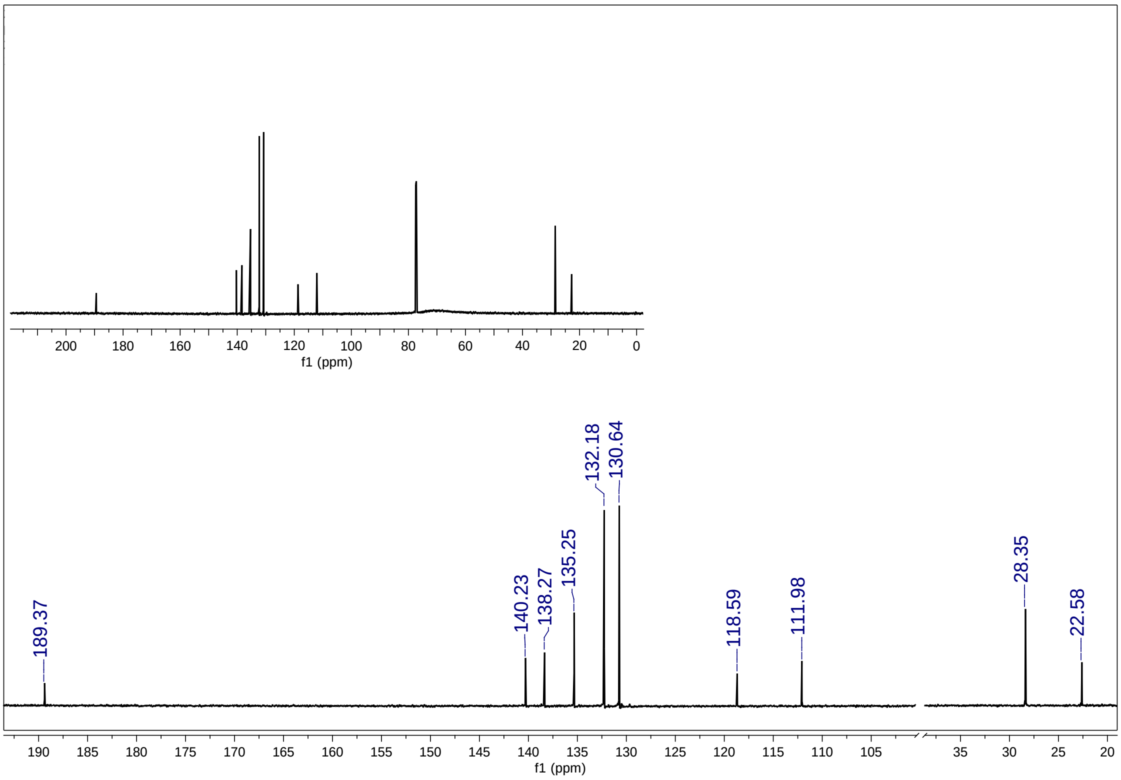

Supplement: Supplementary file 1 [file ijms-19-01909-s001.pdf]
